# Multi-dimensional information characterization of different grades of *Atractylodis macrocephalae* Rhizoma based on HS-GC–MS, LC–MS, electronic nose, and electronic tongue

**DOI:** 10.3389/fnut.2026.1776576

**Published:** 2026-03-25

**Authors:** Ruiqi Yang, Yushi Wang, Jiayu Wang, Ziyue Song, Yunqi Sun, Yuanyu Zhao, Keyao Zhu, Xingyu Guo, Yonghong Yan

**Affiliations:** 1 School of Chinese Materia Medica, Beijing University of Chinese Medicine, Beijing, China; 2 School of Traditional Chinese Materia Medica, Shanxi University of Chinese Medicine, Taiyuan, China

**Keywords:** *Atractylodis macrocephalae* Rhizoma, E-nose, E-tongue, grade classification, HS-GC–MS, LC–MS

## Abstract

*Atractylodes macrocephala* Rhizoma. (AMR, called *Baizhu* in Chinese) is used in traditional Chinese medicine (TCM) for treating gastrointestinal disorders, such as diarrhea and gastritis. With increasing demand, its cultivation areas have expanded significantly. However, the quality of AMR varies considerably by geographical origin and commercial grades, and current research remains insufficient regarding chemical profile differences between grades and rapid classification methods. In this study, we employed Headspace Gas Chromatography–Mass Spectrometry (HS-GC–MS) and Liquid Chromatography-Mass Spectrometry (LC–MS) to analyze volatile and non-volatile components in different grades of AMR. Multivariate statistical methods were applied to elucidate the compositional variation patterns across grades, identify components highly correlated with grading standards. Additionally, the feasibility of applying electronic nose (E-nose) and electronic tongue (E-tongue) technologies for rapid grade classification of AMR was explored. Furthermore, a multi-technology fusion strategy integrating data from HS-GC–MS, LC–MS, E-nose, and E-tongue was implemented to establish a comprehensive grading model the study revealed that there were four volatile differential compounds and six non-volatile differential compounds common to all grades of AMR. Spearman correlation analysis identified terpenoids as the primary volatile compounds contributing to grade-specific aromas, with esters and phenolic acids being key taste compounds. Comparative analysis showed that the multi-technology fusion model, particularly using the Random Forest algorithm, achieved superior classification accuracy (up to 98.33%) compared to models based on any single technology. This study establishes a robust multi-dimensional approach that enhances the quality evaluation research on AMR grading and provides a novel and more reliable strategy for rapid classification of different AMR grades.

## 1 Introduction

*Atractylodes macrocephala* Rhizoma. (AMR) belongs to the family Asteraceae (1), commonly known as *Baizhu* in Chinese, is the dried rhizome. and is used in traditional Chinese medicine (TCM). It has the effects of invigorating the spleen and replenishing qi, drying dampness and promoting diuresis. Clinically, it is often used to treat gastrointestinal disorders (2). The main chemical components of AMR include volatile oils, lactones, polysaccharides, etc. Modern pharmacological research on AMR also focuses on these bioactive compounds, which have been proven to have pharmacological effects such as anti-tumor (3), anti-inflammatory (4), regulating gastrointestinal function (2), and enhancing immune function (5, 6).

The quality of AMR is closely associated with its geographical origin. Zhejiang province, traditionally recognized as a genuine producing region, produces AMR with superior quality, attributed to its specific ecological environment and unique smoking-drying processing method (7, 8). In recent years, due to the pursuit of profits, more and more regions have begun to cultivate AMR, such as Anhui, Henan, Hebei, Hubei, Hunan, and other places. According to field visits and investigations, there are differences in the cultivation years of AMR in each region (e.g., 1 year vs. 2 years) Currently, a significant amount of research is focused on the identification of AMR from different producing regions. For instance, studies have successfully differentiated AMR from regions such as Hubei, Zhejiang, and Hunan through methods including extract-and-shoot inductively coupled plasma mass spectrometry (9). Other approaches involve the construction of dual-channel colorimetric sensors based on metal ion-mediated gold nanoparticle transformations (10) and the application of hyperspectral imaging for discrimination of slices from different origins (11). However, relying solely on tracing geographical origins is considered one-sided, as different cultivation years inevitably lead to differences in medicinal efficacy (12). Therefore, establishing a comprehensive grading system for AMR that integrates origin and cultivation practices is essential. Such a system would not only guide precise clinical application and regulate the market but also promote the sustainable development of the TCM industry.

To establish a scientific grading system, a deep understanding of the chemical composition differences among AMR of different grades is fundamental. Analytical techniques combining chromatography and mass spectrometry are pivotal in TCM component research (13, 14). Headspace gas chromatography–mass spectrometry (HS-GC–MS) integrates headspace sampling with the efficient separation capabilities of gas chromatography and the accurate identification capabilities of mass spectrometry, enabling efficient and accurate qualitative and quantitative analysis of volatile components in samples (15). LC–MS combines the efficient separation capabilities of liquid chromatography with the high sensitivity and selectivity of mass spectrometry, enabling accurate qualitative and quantitative analysis of non-volatile components (16). Applying these techniques to different grades of AMR can elucidate the material basis underlying quality variations. Furthermore, for practical grading and quality control, rapid and bionic discrimination methods are gaining attention. Electronic nose (E-nose) and electronic tongue (E-tongue) technologies, which mimic human sensory evaluation, have shown promise in discriminating TCM materials based on origin or cultivar (17, 18). Their potential for non-destructive, rapid grade classification of AMR warrants exploration.

This study aims to improve the quality evaluation system for AMR by focusing on grade-related chemical differences and rapid discrimination. We employed HS-GC–MS and LC–MS to comprehensively analyze the volatile and non-volatile components in AMR samples from different grades. Multivariate statistical analysis was used to identify key discriminatory components. Additionally, the feasibility of using E-nose and E-tongue for rapid grade classification was evaluated. The integration of these approaches is expected to provide a more robust scientific basis for AMR grading and offer valuable references for its clinical application.

## 2 Materials and methods

### 2.1 Chemical reagents and materials

N-decane (GC, C15862228) and formic acid (HPLC ≥ 98.0%, C15916451) were obtained from Shanghai McLean Biochemical Technology Co., Ltd. (Shanghai, China); Analytical pure n-hexane (20240512) purchased from Tianjin Damao Chemical Reagent Factory (Tianjin China); Analytical pure methanol (240326) purchased from Fuchen Tianjin Chemical Reagent Co., Ltd. (Tianjin, China); Distilled water (241112) purchased from Guangzhou Watsons Food and Beverage Co., Ltd. (Guangdong, China); Acetonitrile (LC–MS, F240BF203) purchased from Thermo Fisher Scientific Co., Ltd. All 39 batches of dried whole tuber AMR samples (numbered ZJ 1 to ZJ 10, AH 1 to AH 13, HN 1 to HN 7, HB 1 to HB 9) were collected from Zhejiang, Anhui, Henan and Hebei province in China, and the source information is listed in the Supplementary Table S1. The authentication of the samples was identified by Professor Yonghong Yan from Department of Chinese Materia Medica of Beijing University of Chinese Medicine according to the morphological features. The voucher specimens are stored in the laboratory of the School of Chinese Materia Medica, Beijing University of Chinese Medicine. All batches of AMR samples were pulverized and sieved through 50 mesh sieve and then stored properly in a refrigerator at −20 °C for further analysis.

### 2.2 Sensory evaluation of AMR

To establish a more objective grading system for AMR, a comprehensive evaluation was performed using fuzzy mathematics. We reviewed extensive literature and consulted identification experts. By integrating insights from the *Pharmacopeia of the People’s Republic of China*, the *Commodity Specification Standards for Seventy-six Medicinal Materials*, and *the Trade Industry Standard of the People’s Republic of China (SB/T 11174–2016)*, along with relevant research studies (19), we ultimately selected sectional color, chrysanthemum pattern, number of oil spots and number per kilogram as descriptive terms for sensory evaluation. The different evaluation criteria were divided into four grades, and the binary comparison determination method was used to determine the proportion of each criterion in the comprehensive evaluation (20). The importance of evaluation factors was compared pairwise, with 1 point assigned to the more important factor and 0 points to the less important one in each comparison. The weight of each of the four indicators was calculated as the ratio of its total score to the sum of all scores, the results are presented in Supplementary Table S2. According to this standard, a total of 10 previously trained professionals were invited to comprehensively evaluate the AMR grades. The number of evaluators for each criterion was counted, and the membership degree matrix as well as the sensory evaluation scores were calculated. The grades were then classified based on the score values. All sensory evaluators are current doctoral or master’s students majoring in Chinese Medicine Identification at Beijing University of Chinese Medicine. To ensure consistency in the evaluation criteria, we conducted specialized training on the identification of AMR prior to the formal experiments. The study was designed and conducted under the guidance and supervision of Professor Yonghong Yan, a specialist in Chinese Medicine Identification.

### 2.3 HS-GC–MS analysis

#### 2.3.1 Solution preparation

N-decane internal standard solution: 100 μL of n-decane was precisely transferred into a 50 mL volumetric flask, dissolved with n-hexane and brought to volume to the mark. Test sample: 1.0 g of sample powder was accurately weighed and placed into a 25 mL headspace vial. And then 20 μL of n-decane internal standard solution was precisely added.

#### 2.3.2 Detection conditions

The analysis was performed using an Agilent 7890B gas chromatography, with a 7697A headspace sampler coupled to an Agilent 5977A mass (Agilent, United States). Chromatography column: HP-5MS capillary column (30 m × 250 μm, 0.25 μm); Injection port temperature: 250 °C; Column chamber temperature: Initial temperature of 50 °C, heated to 100 °C at a rate of 10 °C/min, heated to 220 °C at a rate of 3 °C/min, and heated to 240 °C at a rate of 5 °C/min. Carrier gas: high-purity helium gas; Flow rate: 1 mL/min; Split ratio: 10:1; Injection volume: 1 μL. Interface temperature: 140 °C; Ionization mode: electron bombardment ion source (EI); Ion source temperature: 230 °C; Fourth stage rod temperature: 150 °C; Solvent delay: 3 min; Tuning mode: Standard tuning; Quality scanning range: 40 ~ 450 amu. The equilibrium temperature of headspace sampler was 100 °C, and the equilibrium time was 10 min; Quantitative ring temperature: 120 °C; Transmission line temperature: 140 °C. The compounds obtained by mass spectrometry were identified using the NIST 14.0 database (match threshold > 85%) combined with literature matching (21, 22).

### 2.4 LC–MS analysis

#### 2.4.1 Solution preparation

A precise 1.0 g of AMR powder was weighed and transferred into a 50 mL conical flask. Subsequently, 20 mL of 70% methanol was accurately added, and the total mass was recorded. The mixture was then sonicated for 1 h (250 W, 40 kHz), cooled to room temperature, and reweighed. The mass loss was compensated by replenishing with 70% methanol. After thorough mixing, the solution was centrifuged at 13,000 rpm for 10 min. The supernatant was collected and passed through a 0.22 μm membrane filter, the resulting filtrate was retained as the test sample solution. Additionally, 100 μL aliquots of each test sample solution were pooled, mixed, and prepared as a quality control (QC) sample to monitor the stability of the analytical system and methodology.

#### 2.4.2 Detection conditions

The analysis was performed using an UPLC-Q-Exactive Or-bitrap MS (Thermo Scientific, United States). The chromatographic conditions were as follows: Waters acquity UPLC BEH C18 column (150 mm × 2.1 mm, 1.7 μm), the mobile phase was 0.1% formic acid acetonitrile solution (A) −0.1% formic acid aqueous solution (B); Gradient elution: 0–7 min, 1–40% A; 7–12 min, 40–70% A; 12–16 min, 70–100% A; 16–18 min, 100% A; 18–19 min, 100–1% A; 19–21 min, 1% A; Column temperature 40 °C; Sample manager temperature 10 °C; The volume flow rate was 0.3 mL/min; The injection volume was 3 μL.

Mass Spectrometry Conditions: The system was equipped with an electrospray ionization (ESI) source operating in both positive and negative ion full scan mode. Scan mode: Full MS/dd-MS2; Full MS resolution 70,000; DD MS resolution 17,500; The mass scanning range from m/z 80 to 1,200, the ion transfer tube temperature at 350 °C, the cracking voltage at 3.2 kV, the spray voltage at 3.5 kV, the auxiliary gas temperature at 300 °C, the sheath gas flow rate at 35 L/min, the auxiliary gas flow rate at 15 L/min, and the collision energy at 30 V.

Data Analysis and Compound Identification: Xcalibur 4.1 software was used to preliminary extraction of the collected data, and compound discoverer 3.3 workstation was employed for further data extraction and normalization. Compounds were identified through integrated analysis of retention times, MS^1^ and MS^2^ spectra, and by cross-referencing with fragmentation patterns reported in literature (23, 24) as well as those of reference standards under identical conditions. Additional confirmation was performed using constructed AMR chemical composition database and online databases such as MassBank, PubChem.

### 2.5 E-nose analysis

An *α*-Fox4000 E-nose (Alpha MOS, Co., Ltd., France) equipped with 18 metal-oxide gas sensors was employed to obtain the odor information of AMR. The names and response characteristics of each sensor are presented in Supplementary Table S3. The E-nose was self-checked and the sensor array was preheated for 2–3 h before each sampling experiment. Processed pure air was used as carrier gas to purge the sensor array, making the signal response back to baseline. A single-factor experiment was conducted to investigate the sample amount (0.2, 0.4, 0.6, 0.8 g), incubation temperature (35, 40, 45, 50 °C), incubation time (5, 10, 15 min), and injection volume (500, 1,000, 1,500, 2,000 μL). The optimal parameters were selected based on the maximum value of the sensor response curve and the relative standard deviation (RSD) of the maximum values under each investigated factor. AMR powder (0.4 g) was sealed into a 20 mL vial and incubated for 10 min at 45 °C (250 rpm). The temperature and volume of injection were set at 45 °C and 1,500 μL, respectively. The flow rate of carrier gas was 150 mL/min. The data acquisition interval and data acquisition cycle were set to 1 s and 120 s, respectively. In the experiment, the environmental temperature for E-nose analysis was controlled at 25 °C ± 2 °C. To ensure the reliability of the signals under these conditions, we conducted a repeatability validation of the analysis process. The RSD value for six repeated measurements was below 3%, indicating good repeatability. Every AMR sample was prepared in quadruplicate, and four consecutive injections were performed using an autosampler. The data from the first injection was discarded. The maximum response value from the E-nose sensor in each of the subsequent three injections was selected as the valid data for further analysis.

### 2.6 E-tongue analysis

The ASTREE II E-tongue (Alpha MOS, Co., Ltd., France), equipped with 7 sensors, was employed to obtain the taste information of AMR. The names and response characteristics of each sensor are presented in Supplementary Table S4. 1.0 g of AMR powder was accurately weighed and placed into a 100 mL conical flask. Then, 50 mL of water was added, and the mixture was subjected to ultrasonic extraction for 30 min (250 W, 40 kHz). After extraction, the mixture was centrifuged at 5,000 r/min for 10 min, and the supernatant was collected. The supernatant was passed through a 0.45 μm microporous filter membrane, and the subsequent filtrate was collected as the test solution. The sensors were pre-equilibrated and calibrated using 0.01 mol/L hydrochloric acid. Once pre-equilibration and calibration were successfully completed, testing was initiated. For each sample, a cleaning step (10 s) was applied before acquisition. The sample acquisition time was set to 120 s. In the experiment, the environmental temperature for E-tongue analysis was controlled at 25 °C ± 2 °C. To ensure the reliability of the signals under these conditions, we validated the precision, repeatability, and stability of the electronic tongue analysis process. The precision test results showed that after excluding the first four measurements, the RSD of the sensor response values at each time point was less than 3%. Therefore, in subsequent experiments, each sample was measured 9 times, with the first 4 measurements discarded and the average value of the last 5 measurements was used for data analysis. The RSD value for six repeated measurements was below 3%, indicating good repeatability. The stability test results demonstrated that the RSD of the sensor response values at 120 s over a 12-h period was less than 3%, confirming that the samples remained stable within 12 h.

### 2.7 Data analysis

In order to explore the differential compounds among different grades of AMR, the Orthogonal Partial Least Squares Discriminant Analysis (OPLS-DA) was performed by SIMCA 14.1 software to screen of components with variable importance in projection (VIP) value greater than 1. Similarly, in order to identify which sensors in E-nose and E-tongue have an important contribution to the different grades of AMR, OPLS-DA was also used to analyze and screen the sensors with VIP value greater than 1. SPSS 27.0 software was used to perform Spearman correlation analysis between differential sensors and components. Matlab 2022b software was used to establish the classification models of HS-GC–MS, LC–MS, E-nose and E-tongue, respectively. The trend plots of differential compounds and correlation heat map were drawn by Graphpad prism 9.4.0 software.

## 3 Results and discussion

### 3.1 Sensory evaluation of AMR

An evaluation team consisting of 10 evaluators assessed AMR samples based on sectional color, chrysanthemum pattern, number of oil spots on the section, and number per kilogram. The scores are shown in Supplementary Table S5. AMR from Zhejiang generally received higher scores, mostly concentrated in the range of 70–80. Therefore, as a genuine regional medicinal material, Zhejiang AMR was classified as a separate specification without further grading and was designated as the ZJ group. Anhui AMR is primarily cultivated in Bozhou (33.83°N, 115.78°E). In recent years, Henan has emerged as a major production area for this herb, with its main cultivation regions located in Shangqiu (34.44°N, 115.66°E), Zhoukou (33.58°N, 114.66°E), and other areas. These regions in Henan are geographically close to Anhui in terms of latitude and longitude, share similar processing methods (both employing mechanical drying) and similar morphological appearance of the medicinal material. Therefore, AMR from Anhui and Henan is classified under the same specification. Based on the evaluation scores, samples scoring 60–80 were classified as Grade I, and those scoring 40–60 as Grade II, designated as Group AH-HN-1 and Group AH-HN-2, respectively. Hebei AMR is mainly cultivated using seedlings, supplying 1-year-old seedlings to Anhui and Henan provinces, with the remainder being harvested as medicinal herbs. Given that its cultivation duration and morphological characteristics differ from those of other specifications, Hebei AMR is classified as a separate specification. Based on the evaluation scores, Hebei AMR predominantly scored between 40 and 60 points. Consequently, it was categorized as a distinct specification without further grading and designated as Group HB. The results of the grade classification are presented in Table 1.

**Table 1:** Classification results of sensory evaluation for AMR.

Producing area	Grade	The name of the group	Sample number	Batch
Zhejiang	First class	ZJ	ZJ 1–10	10
Anhui, Henan	Second class	AH-HN-1	AH 1**–**8, HN 1**–**5	13
	Third class	AH-HN-2	AH 9**–**13, HN 6**–**7	7
Hebei	Fourth class	HB	HB 1**–**9	9

### 3.2 Results of HS-GC–MS analysis

**Figure 1:**
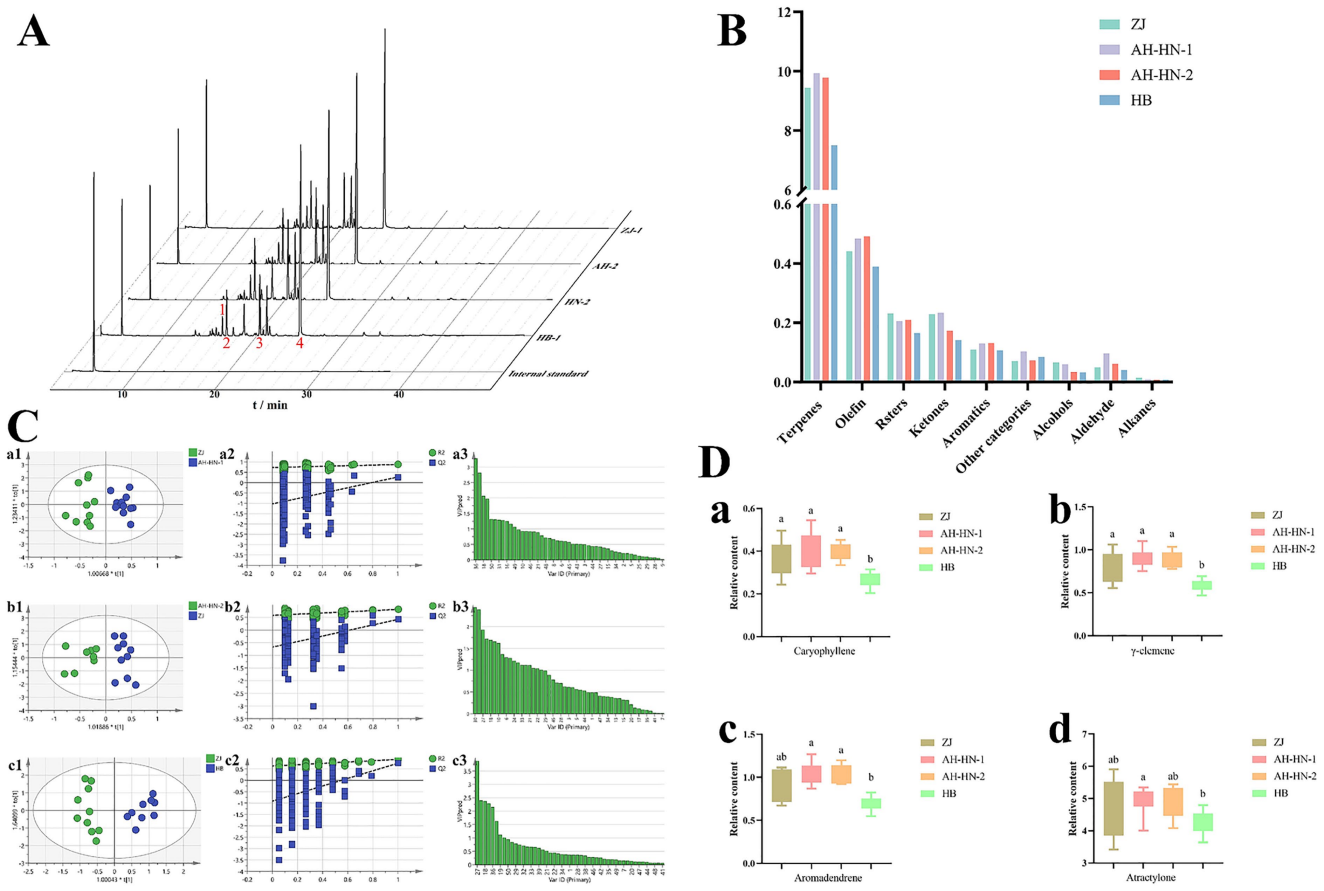
Results of HS-GC–MS analysis. **(A)** Total ion chromatogram of HS-GC–MS. (1: caryophyllene; 2: γ-elemene; 3: aromadendrene; 4: atractylone.) **(B)** Proportional distribution of different compound classes across grades. **(C)** OPLS-DA analysis. (a: ZJ vs. AH-HN-1; b: ZJ vs. AH-HN-2; c: ZJ vs. HB; a1, b1, c1: OPLS-DA score plot; a2, b2, c2: 200 permutation test of OPLS-DA; a3, b3, c3: VIP value of OPLS-DA) **(D)** Box plot of differential compounds (a: caryophyllene; b: γ-elemene; c: aromadendrene; d: atractylone. Different letters indicate significant differences between the two groups, *p <* 0.05).

The total ion chromatogram (TIC) of HS-GC–MS is presented in Figure 1A. The chromatographic profiles of different grades of AMR samples exhibit similar overall patterns. However, variations in peak concentration suggest differences in the abundance of volatile components across grades. A total of 49 chemical constituents were identified in the AMR samples with detailed information provided in Supplementary Table S6. Among these, 22 were shared peaks, and the major components with higher relative abundances include atractylone, (4aR,8aS)-4a-Methyl-1-methylene-7-(propan-2-ylidene) decahydronaphthalene, Aromandendrene, γ-elemene and caryophyllene.

The relative contents of each component were calculated using internal standard peak area as a reference, and the proportional distribution of different compound classes across grades is summarized in Figure 1B. Among the AMR samples, terpenes, alkenes, and ketones are the most abundant classes. Within these three categories, ZJ, AH-HN-1, and AH-HN-2 samples exhibit comparable levels, while HB samples show the lowest contents. This disparity indicates inferior quality of the HB samples.

OPLS-DA could maximizes intergroup differences and helps identify the difference components within massive data set, which is widely employed in differential studies comparing diverse sample groups (25, 26). The OPLS-DA analysis based on the four grades of HS-GC–MS data is shown in Supplementary Figure S1A. The sample points of ZJ (Grade 1) and HB (Grade 4) are completely separated, indicating the greatest difference in volatile composition between them, which is consistent with the significant disparity in sensory evaluation results for their quality. The sample points of AH-HN-1 (Grade 2) and AH-HN-2 (Grade 3) are heavily overlapped, suggesting that the volatile profiles of these two intermediate grades are highly similar and difficult to effectively distinguish by volatile composition alone. Subsequently, the genuine medicinal material ZJ AMR was used as a control for OPLS-DA comparisons with three other grades of samples. The results are shown in Figure 1C, each grade showed an obvious separation trend from ZJ AMR.

In all OPLS-DA models, both *R^2^_X_*(cum) and *R^2^_Y_*(cum) values exceed 0.5, and the permutation test was effective [*R^2^_X_* and R^2^_Y_ represent the explanation rate of the proposed model for the X and Y matrices, respectively, and the closer these indicators are to 1, the more stable and reliable the model (27)]. A total of 25 differential chemical components with VIP > 1 were identified. Among these, four shared differential components are found across comparisons between ZJ and AH-HN-1, ZJ and AH-HN-2, and ZJ and HB samples. These components are atractylone, aromadendrene, γ-elemene, and caryophyllene.

The relative contents of the differential components are shown in Figure 1D. Among them, atractylone has the highest relative content, followed by aromadendrene and γ-elemene, while caryophyllene has the lowest. The relative contents of caryophyllene and γ-elemene in ZJ, AH-HN-1, and AH-HN-2 are higher and are significantly different from those in HB. The relative content of aromadendrene in AH-HN-1 and AH-HN-2 is higher and is significantly different from that in HB. The relative content of atractylone in AH-HN-1 is higher and is significantly different from that in HB. Based on the mean values, the relative contents of the four differential compounds across samples were ranked as follows: AH-HN-1 > AH-HN-2 > ZJ > HB, the trend is generally consistent.

### 3.3 Results of LC–MS analysis

**Figure 2:**
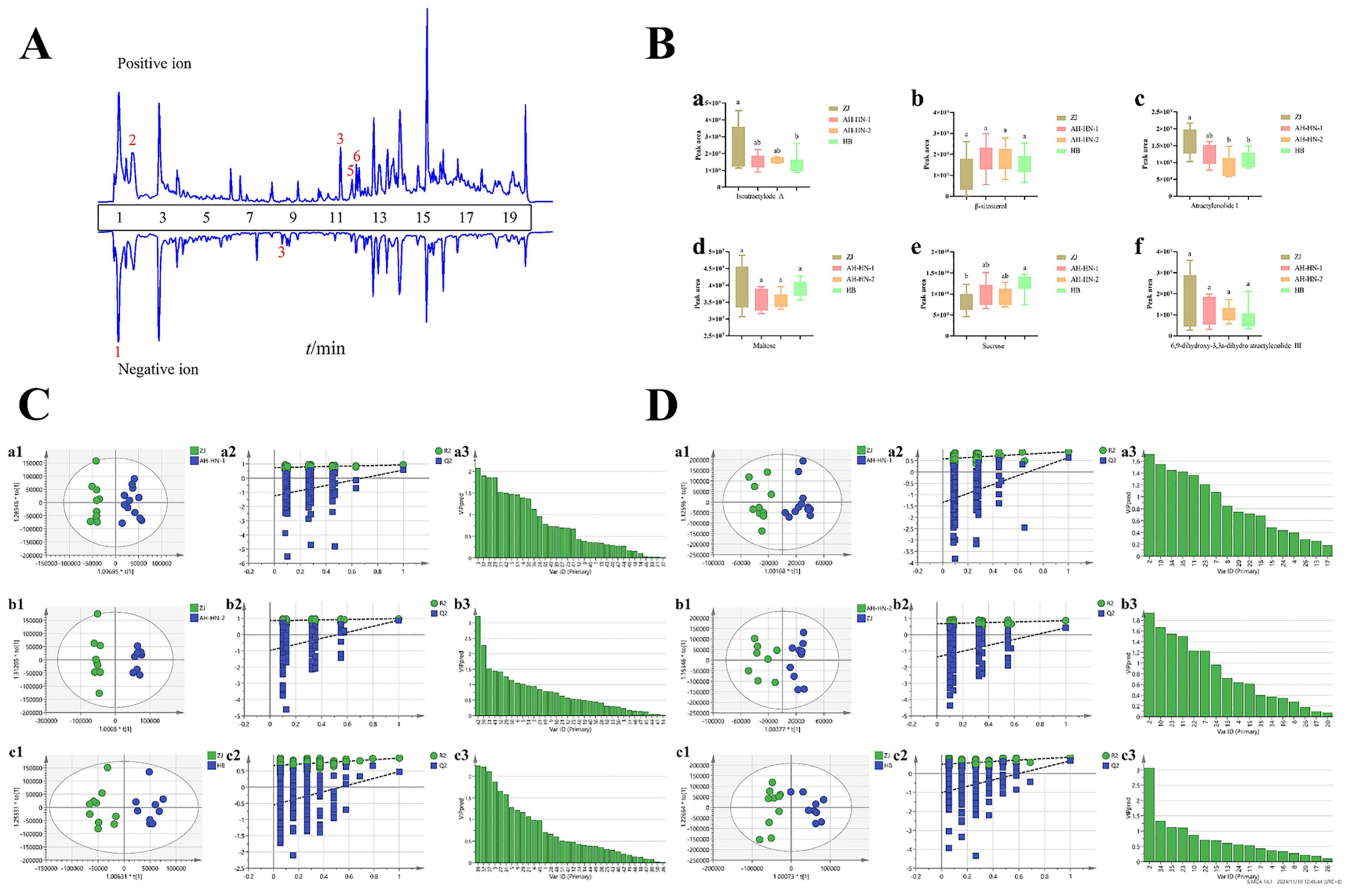
Results of LC–MS analysis **(A)** Representative spectrum of LC–MS. (1: sucrose; 2: maltose; 3: 6,9-dihydroxy-3,3a-dihydro atractylenolide III; 4: β-sitosterol; 5: atractylenolide I; 6: isoatractylode A.) **(B)** Box plot of differential compounds (a: isoatractylode A; b: β-sitosterol; c: atractylenolide I; d: maltose; e: sucrose; f: 6,9-dihydroxy-3,3a-dihydro atractylenolide III. Different letters indicate significant differences between the two groups, *p <* 0.05). **(C)** OPLS-DA analysis of positive ion mode. **(D)** OPLS-DA analysis of negative ion mode (a: ZJ vs. AH-HN-1; b: ZJ vs. AH-HN-2;c: ZJ vs. HB; a1, b1, c1: OPLS-DA score plot; a2, b2, c2: 200 permutation tests of OPLS-DA; a3, b3, c3: VIP value of OPLS-DA).

The representative spectrum of LC–MS is shown in Figure 2A. The overall chromatograms profiles of different grades of AMR are relatively similar. However, differences in peak concentration suggest variations in the content of chemical components among different grades of AMR. Finally, 51 chemical components were identified from AMR, with detailed information provided in Supplementary Table S7.

The OPLS-DA analysis based on the four grades of LC–MS data is shown in Supplementary Figures S1B,C. The tight clustering of QC samples demonstrates the stability of the instrument performance and the good reproducibility of data acquisition throughout the analytical batch, ensuring the reliability of subsequent analysis results. The sample points of ZJ (Grade 1) and HB (Grade 4) are clearly distinguishable, indicating significant differences in non-volatile composition as well. The sample points of AH-HN-1 (Grade 2) and AH-HN-2 (Grade 3) again exhibit overlap, which is consistent with the HS-GC–MS results and further confirms the high similarity in the overall chemical profiles of the intermediate grades, explaining the challenge in distinguishing them using a single chemical dimension. Subsequently, OPLS-DA analysis was also carried out with the genuine medicinal material ZJ AMR as the control and compared with three other grades of samples. The results are shown in Figures 2C,D, indicating that each group shows a significant tendency to separate from ZJ AMR. In the OPLS-DA models, both *R^2^_X_*(cum) and *R^2^_Y_*(cum) values exceed 0.5 across all groups, and the permutation test was effective. A total of 25 differential chemical components with VIP > 1 are identified, including 17 in positive ion mode and 8 in negative ion mode. Among these, six shared differential components were found in comparisons between ZJ and AH-HN-1, ZJ and AH-HN-2, and ZJ and HB samples. These components are isoatractylode A, β-sitosterol, atractylenolide I, maltose, sucrose, and 6,9-dihydroxy-3,3a-dihydro atractylenolide III. Box plots were created with peak areas of each sample as the vertical coordinate, as shown in Figure 2B. The content of isoatractylode A and atractylenolide I was higher in ZJ samples, showing significant differences from HB samples. The content of sucrose was higher in HB samples, showing significant differences from ZJ samples. However, the six components were not strongly correlated with the grade.

Spearman correlation analysis between sensory scores and the aforementioned differential compounds (Supplementary Table S8) revealed that sensory scores were significantly positively correlated with caryophyllene, γ-elemene, aromadendrene, and atractylenolide I. However, in the ZJ samples with higher sensory scores, the contents of compounds like atractylone and isoatractylode A did not increase with sensory scores. The reason may be that atractylone is unstable (28, 29), and the unique smoking-drying method used in Zhejiang promotes the transformation of atractylone into other compounds. Additionally, the sensory characteristics of medicinal materials may be represented by the synergistic effects of multiple components. Therefore, samples with high sensory scores may not necessarily exhibit high contents of every individual compound.

### 3.4 Results of E-nose analysis

**Figure 3:**
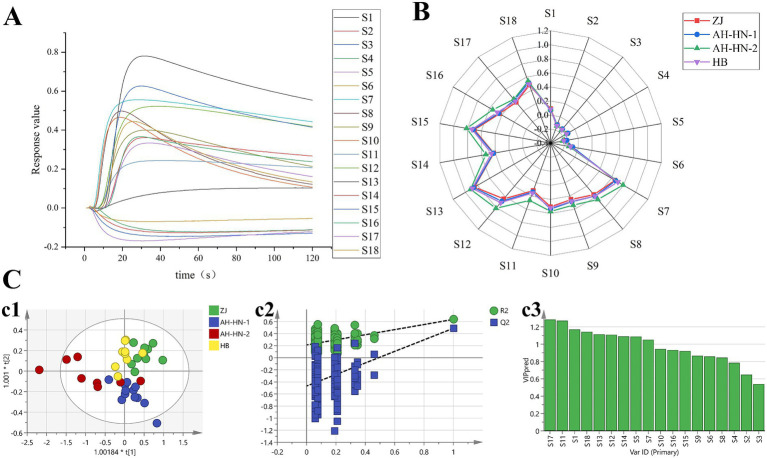
Results of E-nose analysis **(A)** Response values of each sensor (ZJ-1). **(B)** Radar diagram. **(C)** OPLS-DA analysis. (c1: OPLS-DA score plot; c2: 200 permutation tests of OPLS-DA; c3: VIP value of OPLS-DA).

The response values and radar diagram of the sample odor information are shown in Figures 3A,B. It can be observed that the response values of S7–S18 are relatively large, with more pronounced variations in S7–S16. These 10 sensors are particularly sensitive to substances such as organic solvent, hydrocarbons, methane, fluorine, aromatic compounds, ethanol and so on. This suggests that there are differences in odors among different AMR samples, which might be attributed to variations in the types or concentrations of the aforementioned substances.

OPLS-DA analysis was performed to screen for differential sensors of different grades AMR, and the results are shown in Figure 3C. The *R^2^_X_* of 0.997 indicates that the model demonstrates an excellent fit to the sample data. The *R^2^_Y_* of 0.59 and the *Q^2^* of 0.393 reflect the model’s predictive capability, suggesting there is still room for improvement. As shown in Figure 3, the four grades of samples could be generally be distinguished overall. ZJ and HB samples are distributed in the first and second quadrants, exhibiting clear segregation from the other samples, which indicates a significant difference in odor between them and the AH-HN-1 and AH-HN-2 samples. Additionally, AH-HN-1 samples are primarily distributed in the third quadrant, while AH-HN-2 samples are mainly located in the fourth quadrant. Although there is some overlap between the two, they demonstrate a clear trend of separation overall. As can be seen in Figure 3Cc3, there are 9 sensors with VIP values greater than 1, which are ranked in descending order of VIP value as follows: S17, S11, S1, S18, S13, S12, S14, S5, and S7.

Furthermore, Spearman’s rank correlation analysis was conducted between the 9 sensors with significant contributions to classification and the 49 compounds identified by HS-GC–MS (Supplementary Table S9). The results showed that cyclohexene, 3-methyl-6-(1-methylethylidene)- (Cpd 7) had negative correlation with the response values of S7, S11, S12, S13, S14. *α*-Guaiene (Cpd 8), 2-(2R.4aR,8aR)-4a.8-Dimethy/−1.2.3.4.4a,5.6.8a-octahydronaphthalen-2-yprop-2-en-1-ol (Cpd 35) and Cycloprop[e]indene-1a,2(1H)-dicarboxaldehyde, 3a,4,5,6,6a,6b-hexahydro-5,5,6b-trimethyl-, (1a.alpha.,3a.beta.,6a.beta.,6b.alpha.) (Cpd 42) had positive correlation with the response values of S17 and S18. 2-Methylheptanoic acid (Cpd 44) had negative correlation with the response values of S17and S18. Pethylbrene (Cpd 9), modephene (Cpd 10) and (1R,3aS,5aS,8aR)-1,3a,4,5a-Tetramethyl-1,2,3,3a,5a,6,7,8-octahydrocyclopenta[c]pentalene (Cpd 11) had negative correlation with the response values of S5. Compounds 9, 10, and 11 all belong to sesquiterpene hydrocarbons, which are common volatile components in plant essential oils (30, 31). The negative correlation between these three components and sensor S5, which is sensitive to hydrogen sulfide, indicates that these sesquiterpenes may mask or neutralize potential sulfurous odors through their own aromatic properties at the olfactory level. 1H-Cycloprop[e]azulene, 1a,2,3,4,4a,5,6,7b-octahydro-1,1,4,7-tetramethyl- (Cpd 13), isoledene (Cpd 15), Naphthalene, decahydro-4a-methyl-1-methylene-7-(1-methylethenyl)- (Cpd 20) and 1.1.4.7-Tetramethyldecahydro-1H-cyclopropale azulene-4.7-diol (Cpd 38) had positive correlation with the response values of S1. 1-Tridecyne (Cpd 50) had negative correlation with the response values of S1. These compounds (Cpd13, 15, 20, 38) belong to sesquiterpenoids or their oxygenated derivatives. Analysis combining their relative contents (Table S6) reveals that Cpd13, 20, and 38 are present at higher levels in ZJ samples, which may be related to the unique drying method (smoking) employed for ZJ samples. The S1 sensor could specifically detect these oxidation products formed during the pretreatment of AMR. Trimethyl-4-methylenedecahydrocyclopenta [c] pentalene had negative correlation with the response values of S5, and had positive correlation with the response values of S17, S18. (E)-2-((8R,8aS)-8,8a-Dimethyl-3,4,6,7,8,8a-hexahydronaphthalen-2(1H)-ylidene) propyl formate (Cpd 29) and Naphtho[2,3-b]furan-2(4H)-one, 4a,5,6,7,8,8a,9,9a-octahydro-3,8a-dimethyl-5-methylene-(Cpd 48) had positive correlation with the response values of S17. (E)-Valerenyl isovalerate (Cpd 45) had negative correlation with the response values of S11, S12, S14. The results of the correlation analysis between these main volatile flavor compounds and sensor response values provide important clues for an in-depth understanding of the odor characteristics of the samples and their formation mechanisms. The positive and negative correlations between different compounds and the sensors reflect their complex interactions within the samples. Future research could further investigate the interaction mechanisms among these compounds and how they collectively influence the overall odor perception of AMR, thereby offering more profound theoretical support for the quality study of AMR.

### 3.5 Results of E-tongue analysis

**Figure 4:**
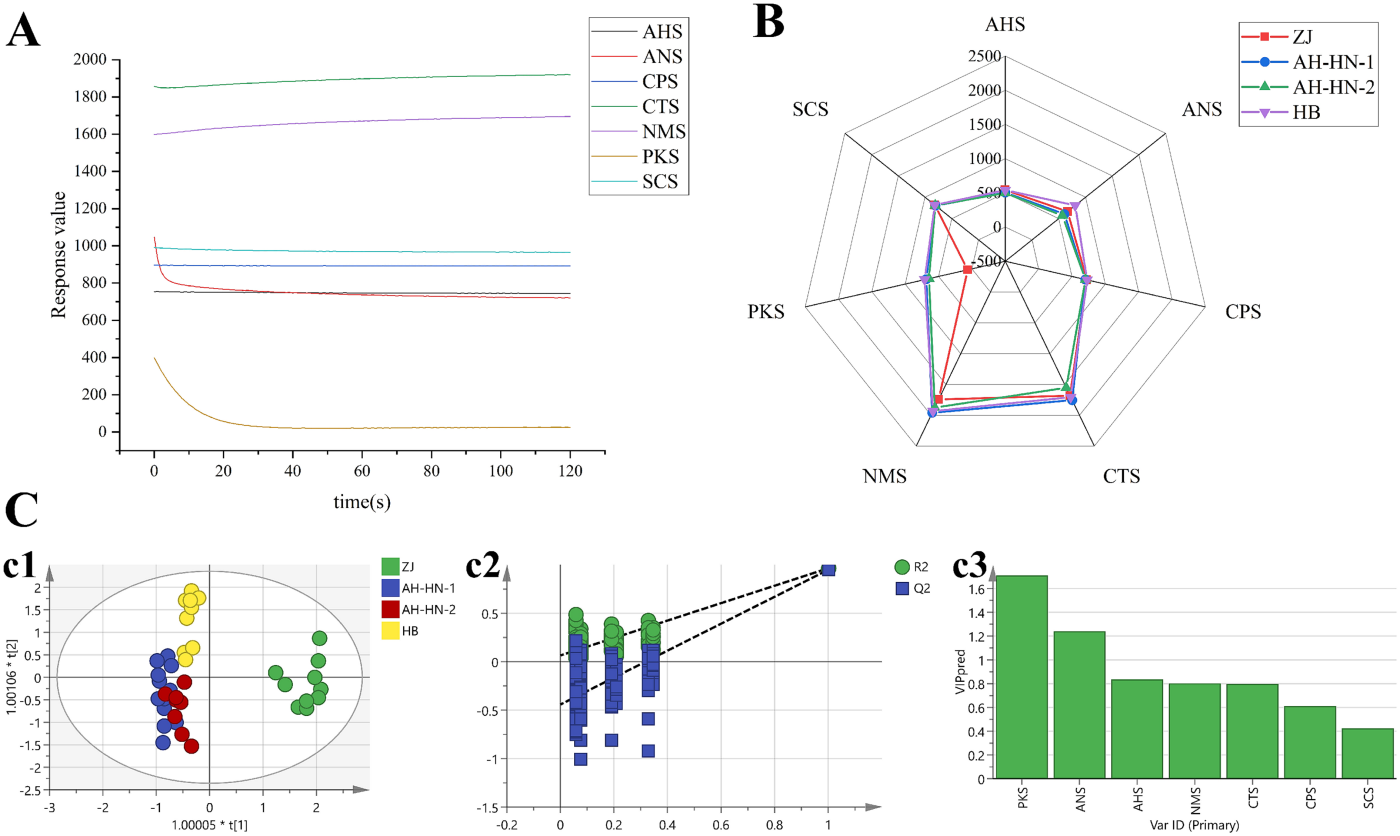
Results of E-tongue analysis **(A)** Response values of each sensor (ZJ-1). **(B)** Radar diagram. **(C)** OPLS-DA analysis. (c1: OPLS-DA score plot; c2: 200 permutation tests of OPLS-DA; c3: VIP value of OPLS-DA).

The response values and radar diagram of the sample taste information are shown in Figures 4A,B. The overall trend of sensors response values is consistent across different grades of AMR, indicating that they have similar taste components. At the same time, differences in the response values can be observed. As can be intuitively seen from the figure, there are slight differences in the taste among the sensors ANS, CTS, NMS, and PKS, with the PKS sensor showing the most pronounced difference between ZJ samples and other grades of samples.

OPLS-DA analysis was performed to screen for differential sensors among different grades AMR, and the results are shown in Figure 4C. The *R^2^_X_* of 0.997 indicates that the model demonstrates an excellent fit to the sample data. The *R^2^_Y_* of 0.614 and the *Q^2^* of 0.492 reflect the model’s predictive capabilities, suggesting there is still room for improvement. As shown in Figure 4c1, ZJ samples are distributed in the first and fourth quadrants, demonstrating distinct separation from AH-HN-1, AH-HN-2, and HB samples. This indicates a distinct taste profiles between ZJ and the other samples. HB samples are distributed in the second quadrant. Although they are close to the AH-HN-1 and AH-HN-2 samples, they could still be distinguished. Consistent with the E-nose OPLS-DA results, the classification challenge primarily resides in distinguishing between AH-HN-1 and AH-HN-2 samples. Figure 4c3 shows that there are 2 sensors with VIP value greater than 1, which are PKS and ANS.

Furthermore, Spearman’s rank correlation analysis was conducted between the 2 sensors with significant contributions to classification and the 51 compounds identified by LC–MS (Supplementary Table S10). The results showed that (E)-deca-2-ene-4,-diyne-1,10-diol-1-O-β-Dapio-furanosyl-(1 → 6)-β-D-glu-copyranoside (Peak1), 6-hydroxyatractylode I (Peak27), 2-Amino-1,3,4-octadecanetriol (Peak30), 9,10-epoxy-12 (z) - octadecenoic acid (Peak34), 1-(4-hydroxy-pentyl)-2,8a-dimethyl-5-methylenedecahy-dro-naphthalene-2,6-diol (Peak35) and 7-[4-(11-hydroxy-undecyloxy)-phenyl]-7-pyridin-3-ylhept-6-enoic acid ethyl ester (Peak37) had negative correlation with the response values of ANS. The negative correlation between these compounds and ANS (sweetness) suggests that they may be key substance groups responsible for masking the sweet taste characteristics in AMR. Neochlorogenic acid (Peak7), 4-(beta-D-Glucosyloxy) benzoate (Peak10), (1R_6R)-6-Hydroxy-2-succinylcyclohexa-2_4-diene-1-carboxylate (Peak11), Isochlorogenic acid B (Peak13), chlorogenic acid (Peak15), 6-(3-hydroxy-propionyloxy) atractylenolid III (Peak20), Rotanane heptaecanate (Peak42), Deoxynaringene (Peak47) and Erucamide (Peak51) had positive correlation with the response values of ANS. Atractylode I (Peak31) had negative correlation with the response values of PKS. 3β-acetoxyatractylone (Peak38) had positive correlation with the response values of PKS. Sucrose (Peak2), L-arginine (Peak3) and Uridine (Peak4) had positive correlation with the response values of ANS and PKS. Sucrose is a direct sweet-tasting substance (32); L-arginine, though alkaline and slightly bitter, also acts as an umami enhancer (33); Uridine is a flavor nucleotide typically associated with umami (34). Their concurrent positive correlations suggest that the synergistic effect between sweetness and umami may serve as an important chemical basis for forming the “sweet” taste profile of Atractylodes macrocephala. DL-Tryptophan (Peak9) and 8,9-epoxy atracolactone (Peak21) had negative correlation with the response values of ANS and PKS. DL-Tryptophan is a bitter amino acid (35), and 8,9-epoxy atractylolactone is a lactone derivative that is likely strongly bitter. Their negative correlations further confirm that bitter components have an antagonistic effect on sweetness and overall palatability. By linking compounds with responses from specific taste sensors, a new perspective is provided for understanding the material basis of the E-tongue discrimination model and for scientifically interpreting the quality differences in AMR from the perspective of “taste.” In the future, these correlations can be further validated by combining artificial sensory evaluations, and the taste interactions among key compounds could be explored in depth.

### 3.6 Classification model for different grades of AMR

Three commonly used machine learning algorithms—K-Nearest Neighbors (KNN), Backpropagation Neural Network (BPNN), and Random Forest (RF)—were employed to develop AMR grade classification models based on HS-GC–MS, LC–MS, E-nose and E-tongue, respectively. Principal Component Analysis (PCA) was applied to extract the principal components with a cumulative contribution rate of 95% from HS-GC–MS, LC–MS, E-nose and E-tongue data, which were then used as the input layer. Additionally, the principal component feature layers from the four techniques were also employed as the fused data for model input. 70% of the data was randomly selected as the training set, with 30% of the data used as the testing set. The results are presented in Table 2.

**Table 2:** Accuracy of the AMR grade classification models.

Data source	Classifier	Training set	Testing set
HS-GC–MS	KNN	85.19%	85.00%
	BPNN	81.48%	63.33%
	RF	100.0%	85.00%
LC–MS	KNN	78.52%	70.00%
	BPNN	83.70%	68.33%
	RF	100.0%	73.33%
E-nose	KNN	66.67%	73.33%
	BPNN	68.15%	65.00%
	RF	100.0%	73.33%
E-tongue	KNN	81.48%	91.67%
	BPNN	83.71%	88.33%
	RF	100.0%	93.34%
Fusion data	KNN	92.59%	91.67%
	BPNN	90.37%	76.67%
	RF	100.00%	98.33%

Among the single-technology models, KNN and RF algorithms demonstrated better predictive performance. The classification model based on the E-tongue performed the best, with a testing set accuracy greater than 90.0%. The KNN and RF models based on HS-GC–MS achieved an accuracy of 85.0%, indicating better predictive performance. However, the accuracy of the classification models based on LC–MS and E-nose were less than 80.0%. The high performance of the E-tongue classification model may be attributed to its more comprehensive capture of taste information from AMR samples, thereby providing a richer and more detailed data foundation for the classification model. Compared to HS-GC–MS and LC–MS, the data from the E-tongue may more directly reflect the taste attributes of the samples and are more relevant to the grade classification objectives of AMR. The E-nose is primarily used to detect odor characteristics of samples, since the odor of AMR samples is affected by drying temperature and time, it may lead to low accuracy of classification model. Additionally, this study extracted the maximum response value of the E-nose sensors as a feature for classification. It is possible that the maximum value alone does not fully capture the information of AMR samples. Future research could explore extracting other features, such as the integral value of the response curve or the steady-state value, for analysis to improve the accuracy of the classification model.

Although KNN and RF algorithms achieved a certain accuracy (85.0%) in the HS-GC–MS model, it may fail to achieve higher prediction performance due to the impact of data complexity and noise. LC–MS is mainly used to analyze non-volatile compounds. However, such compounds in AMR may be diverse, present in low concentrations, and significantly affected by sample matrix interference, resulting in poor data quality and consequently high complexity and noise (36). The model may struggle to adequately learn effective classification features, leading to poor generalization ability and an accuracy below 80.0%.

A comparison between the “single-technology model” and the “multi-technology fusion model” revealed that the fusion model achieved superior performance on the testing set. For the KNN algorithm, the accuracy of the fusion model (91.67%) was significantly higher than that of three single-technology models (HS-GC–MS: 85.00%, LC–MS: 70.00%, E-nose: 73.33%) and matched the best-performing single-technology model (E-tongue: 91.67%). This demonstrates that the fusion approach enhances the discriminative power of weaker technologies without compromising the advantages of E-tongue. For the RF algorithm, the accuracy of the fusion model reached 98.33%, which is not only the highest among all models but also surpasses all single-technology RF models. This confirms that multi-source information fusion can overcome the limitations of individual technologies and achieve improved classification performance to a certain extent (37). For the BPNN algorithm, the performance of the fusion model falls between that of the individual single-technology models.

As the data demonstrate, the E-tongue technology exhibited outstanding performance in the grade discrimination of AMR in this study, highlighting the strong potential of taste fingerprint as a dimension for quality evaluation of AMR. However, the accuracy of 98.33% achieved by the feature fusion model, particularly with the RF algorithm, is not a simple summation of the performances of individual technologies. This proves that there exists strong complementarity among the multi-dimensional information derived from chemical composition (HS-GC–MS, LC–MS) and overall sensory attributes (E-nose, E-tongue). The fusion model can comprehensively utilize the correlations and differences between “chemical specificity” and “holistic sensory fingerprints,” thereby enabling more precise discrimination of herbal grades that aligns more closely with the integrated judgment of human experts (combining appearance, color, aroma, and taste). “Multi-dimensional Information Characterization” constructs a feature space with richer information by integrating data from different technological platforms, allowing for a more detailed characterization of the complex and subtle differences among various grades of AMR.

Furthermore, the machine learning models developed in this study employed default parameters (e.g., the number of nearest neighbors *K* = 2 in the KNN model; for the BPNN model, the number of hidden layers was 1, the number of neurons in the hidden layer was 5, and the maximum number of iterations was 1,000; for the RF model, ntree was 500, mtry was 3), which may be one of the factors limiting classification accuracy. The next step will focus on systematic optimization of the model parameters and explore more advanced ensemble learning or deep learning architectures to establish a more robust classification model for Atractylodes macrocephala grades.

However, regardless of the analytical method or machine learning algorithm used, the classification difficulty primarily lies in distinguishing between the AH-HN-1 and AH-HN-2 groups. We further analyzed the differences between these groups and found that after sensory evaluation-based grading: All samples from the ZJ group were classified as Grade 1, with a growth age of 2 years; All samples from the HB group were classified as Grade 4, with a growth age of 1 year; All samples from the AH-HN-1 group were classified as Grade 2, with a growth age of 2 years; In the AH-HN-2 group (classified as Grade 3), only two batches had a growth age of 2 years, while the rest were 1 year. The confusion observed in the AH-HN-2 group may explain its low classification accuracy. In future studies, we will collect more samples featuring “same origin, different ages” and “same age, different origins” and apply two-way ANOVA to draw definitive conclusions.

## 4 Conclusion

This study collected AMR from four different regions and classified them into grades based on sensory evaluation. Firstly, the differential volatile and non-volatile compounds in AMR of different grades were identified by HS-GC–MS and LC–MS combined with chemometrics. The results indicated that the quality of samples from Zhejiang, Anhui, and Henan was relatively good, while that from Hebei was the poorest. Current quality control standards for AMR, such as the *Chinese Pharmacopeia* (1), only include the content of 60% ethanol-soluble extract as a quality indicator. In most reported studies (28, 38, 39), the primary compounds used for identification are atractylone and atractylenolides. Based on the findings of this study, further research can be conducted on the differential volatile compound atractylone and the non-volatile differential compound atractylenolide I. Expanding the sample size to investigate their variation trends across different grades and establishing content limits could provide a reference for improving the quality control of AMR.

Subsequently, the odor and taste profiles of AMR were characterized using E-nose and E-tongue technologies. Furthermore, terpene components were identified as the main volatile compounds contributing to the odor of different grades through Spearman correlation analysis, while ester and phenolic acid components were identified as key flavor compounds. While the E-tongue demonstrated high classification accuracy in this study, we acknowledge that this result is based on a specific sample set and algorithm. Further validation with larger and more diverse samples, as well as in practical application scenarios, is necessary to confirm its robustness and generalizability.

To achieve a more comprehensive and reliable evaluation, this study further explored a multi-technology fusion strategy. The integration of features from HS-GC–MS, LC–MS, E-nose, and E-tongue data was used to construct a multi-dimensional representation model. Comparative analysis revealed that the fusion model, particularly when employing the RF algorithm, achieved superior and more robust classification performance compared to models based on any single technology. These results demonstrate that integrating chemical composition data with holistic sensory fingerprints provides a more informative feature space, enabling a more nuanced discrimination of AMR grades. This multi-technology approach offers a complementary and potentially more powerful strategy for AMR grade classification.

In summary, this study provides a preliminary yet promising strategy for the AMR grade classification. The research fills the gap in the study of differences in volatile and non-volatile components among different grades of AMR, enriches the research on quality evaluation of AMR, and also provides a reference for the quality control of AMR. Future work should focus on expanding the sample size, validating the model in practical settings, and systematically optimizing the fusion methodology to enhance its applicability and reliability.

## Data Availability

The original contributions presented in the study are included in the article/Supplementary material, further inquiries can be directed to the corresponding authors.
