# Supplementary material for: Multi-dimensional information characterization of different grades of Atractylodis macrocephalae Rhizoma based on HS-GC–MS, LC–MS, electronic nose, and electronic tongue
Source: Front Nutr. 2026 Mar 25;13:1776576. doi: 10.3389/fnut.2026.1776576 (PMC13057392; doi:10.3389/fnut.2026.1776576)
Supplement: Supplementary file 1 [file Supplementary_file_1.docx]

**Multi-dimensional Information Characterization of Different Grades of *Atractylodis macrocephalae* Rhizoma Based on HS-GC-MS, LC-MS, Electronic Nose, and Electronic Tongue**

Ruiqi Yang^1,2,†,*^ Yushi Wang^1, †^, Jiayu Wang^1^, Ziyue Song^1^, Yunqi Sun^1^, Yuanyu Zhao^1^, Keyao Zhu^1^, Xingyu Guo^1^, Yonghong Yan^1*^

1 School of Chinese Materia Medica, Beijing University of Chinese Medicine, Beijing, 102401, China.

2 School of Traditional Chinese Materia Medica, Shanxi University of Chinese Medicine, Taiyuan 030619, China.

* Correspondence: yangruiqi@sxtcm.edu.cn (R.Y.); lxdyyh@yeah.net (Y.Y.).

† These authors contributed equally to this work.

## Table S1

Table S1 The AMR samples information

| Sample Number | Origin | Growth years |
| --- | --- | --- |
| ZJ-1 | Pan'an Traditional Chinese Medicine Market, Pan'an County, Jinhua City, Zhejiang Province | 2 |
| ZJ-2 | Baixuebei Village, Dapan Town, Pan'an County, Jinhua City, Zhejiang Province | 2 |
| ZJ-3 | Shuangfeng Township, Pan'an County, Jinhua City, Zhejiang Province | 2 |
| ZJ-4 | Yiaochuan Township, Pan'an County, Jinhua City, Zhejiang Province | 2 |
| ZJ-5 | Tantou Town, Tiantai County, Taizhou City, Zhejiang Province | 2 |
| ZJ-6 | Dachengu, Butou Town, Xianju County, Taizhou City, Zhejiang Province | 2 |
| ZJ-7 | Xiawang Village, Xinchang County, Shaoxing City, Zhejiang Province | 2 |
| ZJ-8 | Honglian Village, Huishan Town, Xinchang County, Shaoxing City, Zhejiang Province | 2 |
| ZJ-9 | Wangjiashi Village, Huishan Town, Xinchang County, Shaoxing City, Zhejiang Province | 2 |
| ZJ-10 | Yujiang Village, Ru'ao Town, Xinchang County, Shaoxing City, Zhejiang Province | 2 |
| AH-1 | Xinji Village, Qiaodong Town, Qiaocheng District, Bozhou City, Anhui Province | 2 |
| AH-2 | Wuma Town, Bozhou City, Anhui Province | 2 |
| AH-3 | Hua Tuo Town, Qiaocheng District, Bozhou City, Anhui Province | 2 |
| AH-4 | Weigang Town, Qiaocheng District, Bozhou City, Anhui Province | 2 |
| AH-5 | Shibali Town, Qiaocheng District, Bozhou City, Anhui Province | 2 |
| AH-6 | Shibali Town, Qiaocheng District, Bozhou City, Anhui Province | 2 |
| AH-7 | Niuji Town, Qiaocheng District, Bozhou City, Anhui Province | 2 |
| AH-8 | Caozhuang Village, Niuji Town, Qiaocheng District, Bozhou City, Anhui Province | 2 |
| AH-9 | Tongguan Village, Qiaodong Town, Qiaocheng District, Bozhou City, Anhui Province | 2 |
| AH-10 | Weigang Town, Qiaocheng District, Bozhou City, Anhui Province | 2 |
| AH-11 | Wuma Town, Qiaocheng District, Bozhou City, Anhui Province | 1 |
| AH-12 | Wuma Town, Qiaocheng District, Bozhou City, Anhui Province | 1 |
| AH-13 | Hua Tuo Town, Qiaocheng District, Bozhou City, Anhui Province | 1 |
| HN-1 | Dancheng, Zhoukou City, Henan Province | 2 |
| HN-2 | Dancheng, Zhoukou City, Henan Province | 2 |
| HN-3 | Yucheng County, Shangqiu City, Henan Province | 2 |
| HN-4 | Yucheng County, Shangqiu City, Henan Province | 2 |
| HN-5 | Yucheng County, Shangqiu City, Henan Province | 2 |
| HN-6 | Dancheng, Zhoukou City, Henan Province | 1 |
| HN-7 | Dancheng, Zhoukou City, Henan Province | 1 |

Table S1 The AMR samples information (Continued)

| Sample Number | Origin | Growth years |
| --- | --- | --- |
| HB-1 | Chencunying Village, Dingzhou City, Hebei Province | 1 |
| HB-2 | Chencunying Village, Dingzhou City, Hebei Province | 1 |
| HB-3 | Daluzhuang Village, Dingzhou City, Hebei Province | 1 |
| HB-4 | Xin'an Village, Zhengzhang Town, Anguo City, Hebei Province | 1 |
| HB-5 | Xikuang Village, Zhengzhang Town, Anguo City, Hebei Province | 1 |
| HB-6 | Beidu Village, Beiduan Village Township, Anguo City, Hebei Province | 1 |
| HB-7 | Anguo City, Hebei Province | 1 |
| HB-8 | Anguo City, Hebei Province | 1 |
| HB-9 | Anguo City, Hebei Province | 1 |

## Table S2

Table S2 Weight of sensory evaluation indicators

| Evaluation metric | Score of each indicator | | | | | Weight |
| --- | --- | --- | --- | --- | --- | --- |
|  | Sectional color | Number of oil spots | Ehrysanthemum pattern | Number per kilogram | Total |  |
| Sectional color | 10 | 3 | 5 | 3 | 21 | 0.21 |
| Number of oil spots | 7 | 10 | 8 | 7 | 32 | 0.32 |
| Ehrysanthemum pattern | 5 | 2 | 10 | 3 | 20 | 0.20 |
| Number per kilogram | 7 | 3 | 7 | 10 | 27 | 0.27 |

## Table S3

Table S3 Detailed information of 18 metal oxide sensors

| Number | Type of sensors | Sensitive substance |
| --- | --- | --- |
| S1 | LY2/LG | Oxidizing gas |
| S2 | LY2/G | Ammonia/organic amines, carbon monoxide |
| S3 | LY2/AA | Ethanol |
| S4 | LY2/GH | Ammonia/organic amines |
| S5 | LY2/gCTL | Hydrogen sulfide |
| S6 | LY2/gCT | Propane, butane |
| S7 | T30/1 | Organic solvent |
| S8 | P10/1 | Hydrocarbons, methane |
| S9 | P10/2 | Methane |
| S10 | P40/1 | Fluorine |
| S11 | T70/2 | Aromatic compounds |
| S12 | PA/2 | Ethanol, ammonia/organic amines |
| S13 | P30/1 | Polar compound (ethanol) |
| S14 | P40/2 | Heteroatoms/chlorides/aldehydes |
| S15 | P30/2 | Alcohol |
| S16 | T40/2 | Aldehyde compounds |
| S17 | T40/1 | Chlorinated compounds |
| S18 | TA/2 | Air |

## Table S4

Table S4 Detailed information of 7 E-tongue sensors

| Number | Type of sensors | Sensitive substance |
| --- | --- | --- |
| D1 | AHS | Sourness |
| D2 | PKS | General purpose |
| D3 | CTS | Saltiness |
| D4 | NMS | Umami |
| D5 | CPS | General purpose |
| D6 | ANS | Sweetness |
| D7 | SCS | Bitterness |

## Table S5

Table S5 The score values of sensory evaluation

| Sample Number | Score value | Sample Number | Score value |
| --- | --- | --- | --- |
| ZJ-1 | 74.32 | AH-1 | 64.16 |
| ZJ-2 | 74.12 | AH-2 | 70.28 |
| ZJ-3 | 73.20 | AH-3 | 70.76 |
| ZJ-4 | 72.38 | AH-4 | 78.16 |
| ZJ-5 | 78.14 | AH-5 | 65.72 |
| ZJ-6 | 73.86 | AH-6 | 67.88 |
| ZJ-7 | 70.12 | AH-7 | 69.92 |
| ZJ-8 | 76.68 | AH-8 | 71.72 |
| ZJ-9 | 75.16 | AH-9 | 48.44 |
| ZJ-10 | 73.62 | AH-10 | 58.04 |
| HB-1 | 57.02 | AH-11 | 50.76 |
| HB-2 | 56.20 | AH-12 | 59.60 |
| HB-3 | 58.70 | AH-13 | 59.60 |
| HB-4 | 46.24 | HN-1 | 64.32 |
| HB-5 | 48.48 | HN-2 | 64.32 |
| HB-6 | 53.78 | HN-3 | 64.52 |
| HB-7 | 53.80 | HN-4 | 67.08 |
| HB-8 | 42.42 | HN-5 | 65.80 |
| HB-9 | 43.26 | HN-6 | 45.18 |
|  |  | HN-7 | 45.58 |

## Table S6

Table S6 Results of HS-GC-MS for AMR

| Serial Number | Retention Time | Compound | molecular formula | Average Relative Content（%） | | | |
| --- | --- | --- | --- | --- | --- | --- | --- |
|  |  |  |  | ZJ | AH-HN-1 | AH-HN-2 | HB |
| 1 | 3.556 | Butanoic acid, 3-methyl- | C_5_H_10_O_2_ | 0.0124 | 0.0049 | 0.0057 | 0.0027 |
| 2 | 3.77 | Tert-butyl N-benzylcarbamate | C_12_H_17_NO_2_ | 0.0071 | 0.0051 | 0.0063 | 0.0039 |
| 3 | 3.829 | Tricyclo[2.2.2.0(1,4)]octane | C_8_H_12_ | 0.0074 | 0.0030 | 0.0000 | 0.0020 |
| 4 | 5.39 | Decane | C_10_H_22_ | 1.0000 | 1.0000 | 1.0000 | 1.0000 |
| 5 | 13.124 | Silphiperfol-5-ene | C_15_H_24_ | 0.0148 | 0.0159 | 0.0171 | 0.0128 |
| 6 | 13.337 | Bicyclo[2.2.1]heptan-2-one, 1-ethenyl-7,7-dimethyl- | C_11_H_16_O | 0.0881 | 0.0778 | 0.0594 | 0.0546 |
| 7 | 13.498 | Cyclohexene, 3-methyl-6-(1-methylethylidene)- | C_10_H_16_ | 0.0028 | 0.0022 | 0.0027 | 0.0006 |
| 8 | 13.759 | α-Guaiene | C_15_H_24_ | 0.0436 | 0.0529 | 0.0545 | 0.0382 |
| 9 | 14.78 | Pethylbrene | C_15_H_24_ | 0.0068 | 0.0065 | 0.0078 | 0.0063 |
| 10 | 14.946 | Modephene | C_15_H_24_ | 0.0802 | 0.0985 | 0.1006 | 0.0758 |
| 11 | 15.171 | (1R,3aS,5aS,8aR)-1,3a,4,5a-Tetramethyl-1,2,3,3a,5a,6,7,8-octahydrocyclopenta[c]pentalene | C_15_H_24_ | 0.1085 | 0.1304 | 0.1317 | 0.1069 |
| 12 | 15.338 | 1,5-Cyclodecadiene, 1,5-dimethyl-8-(1-methylethenyl)-, [S-(Z,E)]- | C_15_H_24_ | 0.0758 | 0.0695 | 0.0647 | 0.0416 |
| 13 | 15.593 | 1H-Cycloprop[e]azulene, 1a,2,3,4,4a,5,6,7b-octahydro-1,1,4,7-tetramethyl-, [1aR-(1a.alpha.,4.alpha.,4a.beta.,7b.alpha.)]- | C_15_H_24_ | 0.1119 | 0.0959 | 0.1138 | 0.0920 |
| 14 | 15.848 | (1R,3aS,5aS,8aR)-1,3a,5a-Trimethyl-4-methylenedecahydrocyclopenta[c]pentalene | C_15_H_24_ | 0.0588 | 0.0828 | 0.0800 | 0.0568 |

Table S6 Results of HS-GC-MS for AMR(Continued)

| Serial Number | Retention Time | Compound | Molecular formula | Average Relative Content（%） | | | |
| --- | --- | --- | --- | --- | --- | --- | --- |
|  |  |  |  | ZJ | AH-HN-1 | AH-HN-2 | HB |
| 15 | 16.062 | isoledene | C_15_H_24_ | 0.0059 | 0.0030 | 0.0048 | 0.0040 |
| 16 | 16.275 | **Caryophyllene** | C_15_H_24_ | 0.3741 | 0.4097 | 0.4035 | 0.2669 |
| 17 | 16.447 | 1,5-Cyclodecadiene, 1,5-dimethyl-8-(1-methylethylidene)-, (E,E)- | C_15_H_24_ | 0.0186 | 0.0198 | 0.0173 | 0.0133 |
| 18 | 16.732 | **γ-Elemene** | C_15_H_24_ | 0.8200 | 0.8985 | 0.8819 | 0.5971 |
| 19 | 17.445 | Humulene | C_15_H_24_ | 0.1733 | 0.1817 | 0.1797 | 0.1224 |
| 20 | 18.175 | Naphthalene, decahydro-4a-methyl-1-methylene-7-(1-methylethenyl)-, [4aR-(4a.alpha.,7.alpha.,8a.beta.)]- | C_15_H_24_ | 0.0275 | 0.0259 | 0.0308 | 0.0256 |
| 21 | 18.347 | 1-Methyl-4-(6-methylhept-5-en-2-yl)cyclohexa-1,3-diene | C_15_H_24_ | 0.0257 | 0.0377 | 0.0376 | 0.0380 |
| 22 | 18.477 | (R,Z)-2-Methyl-6-(4-methylcyclohexa-1,4-dien-1-yl)hept-2-en-1-ol | C_15_H_24_O | 0.0342 | 0.0249 | 0.0112 | 0.0166 |
| 23 | 18.614 | Naphthalene, 1,2,3,5,6,7,8,8a-octahydro-1,8a-dimethyl-7-(1-methylethenyl)-, [1S-(1.alpha.,7.alpha.,8a.alpha.)]- | C_15_H_24_ | 0.6507 | 0.6211 | 0.6088 | 0.4111 |
| 24 | 18.911 | 1,3-Cyclohexadiene, 5-(1,5-dimethyl-4-hexenyl)-2-methyl-, [S-(R*,S*)]- | C_15_H_24_ | 0.0336 | 0.0470 | 0.0508 | 0.0438 |
| 25 | 19.771 | Naphthalene, 1,2,3,5,6,7,8,8a-octahydro-1,8a-dimethyl-7-(1-methylethenyl)-, [1R-(1.alpha.,7.beta.,8a.alpha.)]- | C_15_H_24_ | 0.0575 | 0.0569 | 0.0550 | 0.0390 |
| 26 | 19.932 | Cyclohexene, 3-(1,5-dimethyl-4-hexenyl)-6-methylene-, [S-(R*,S*)]- | C_15_H_24_ | 0.0276 | 0.0463 | 0.0425 | 0.0460 |
| 27 | 20.317 | (4aR,8aS)-4a-Methyl-1-methylene-7-(propan-2-ylidene)decahydronaphthalene | C_15_H_24_ | 1.4484 | 1.4042 | 1.3170 | 0.8327 |
| 28 | 20.543 | Selina-3,7(11)-diene | C_15_H_24_ | 0.0200 | 0.0198 | 0.0283 | 0.0119 |
| 29 | 20.703 | (E)-2-((8R,8aS)-8,8a-Dimethyl-3,4,6,7,8,8a-hexahydronaphthalen-2(1H)-ylidene)propyl formate | C_16_H_24_O_2_ | 0.1072 | 0.1077 | 0.1263 | 0.0790 |

Table S6 Results of HS-GC-MS for AMR(Continued)

| Serial Number | Retention Time | Compound | Molecular formula | Average Relative Content（%） | | | |
| --- | --- | --- | --- | --- | --- | --- | --- |
|  |  |  |  | ZJ | AH-HN-1 | AH-HN-2 | HB |
| 30 | 21.095 | **Aromandendrene** | C_15_H_24_ | 0.9233 | 1.0359 | 1.0306 | 0.7059 |
| 31 | 21.391 | (8R,8aS)-8,8a-Dimethyl-2-(propan-2-ylidene)-1,2,3,7,8,8a-hexahydronaphthalene | C_15_H_22_ | 0.1852 | 0.2094 | 0.2070 | 0.1469 |
| 32 | 22.104 | 1,3-Bis-(2-cyclopropyl,2-methylcyclopropyl)-but-2-en-1-one | C_18_H_26_O | 0.0561 | 0.0477 | 0.0328 | 0.0352 |
| 33 | 23.101 | 1,3,3-Trimethyl-2-(2-methyl-cyclopropyl)-cyclohexene | C_13_H_22_ | 0.0273 | 0.0133 | 0.0098 | 0.0064 |
| 34 | 23.683 | 1H-Cycloprop[e]azulen-7-ol, decahydro-1,1,7-trimethyl-4-methylene-, [1ar-(1a.alpha.,4a.alpha.,7.beta.,7a.beta.,7b.alpha.)]- | C_15_H_24_O | 0.0147 | 0.0129 | 0.0107 | 0.0077 |
| 35 | 24.252 | 2-(2R.4aR,8aR)-4a.8-Dimethy/-1.2.3.4.4a,5.6.8a-octahydronaphthalen-2-yprop-2-en-1-ol | C_15_H_24_O | 0.0024 | 0.0060 | 0.0025 | 0.0000 |
| 36 | 24.745 | **Atractylon** | C₁₅H₂₀O | 4.6733 | 4.9720 | 4.9335 | 4.2618 |
| 37 | 25.072 | (4aS,8aR)-3,8a-Dimethyl-5-methylene-4,4a,5,6,7,8,8a,9-octahydronaphtho[2,3-b]furan | C_15_H_20_O | 0.0119 | 0.0083 | 0.0078 | 0.0096 |
| 38 | 26.917 | 1.1.4.7-T etramethyldecahydro-1H-cyclopropale]azulene-4.7-diol | C_25_H_24_O | 0.0123 | 0.0108 | 0.0069 | 0.0071 |
| 39 | 27.285 | 2H-Cyclopropa[a]naphthalen-2-one, 1,1a,4,5,6,7,7a,7b-octahydro-1,1,7,7a-tetramethyl-, (1a.alpha.,7.alpha.,7a.alpha.,7b.alpha.)- | C_15_H_22_O | 0.0656 | 0.0728 | 0.0606 | 0.0386 |
| 40 | 28.668 | Acetic acid, hydrazide | C_2_H_6_N_2_O | 0.0129 | 0.0139 | 0.0086 | 0.0074 |
| 41 | 30.847 | (E)-2-(8R.8aS)-8.8a-Dimethyl-3.4,6.7.8.8a-hexahydronaphthalen-2(1H)-ylidene)propan-1-ol | C_15_H_24_O | 0.0025 | 0.0059 | 0.0025 | 0.0017 |
| 42 | 31.648 | Cycloprop[e]indene-1a,2(1H)-dicarboxaldehyde, 3a,4,5,6,6a,6b-hexahydro-5,5,6b-trimethyl-, (1a.alpha.,3a.beta.,6a.beta.,6b.alpha.)-(+)- | C_15_H_20_O_2_ | 0.0500 | 0.0959 | 0.0617 | 0.0403 |
| 43 | 33.351 | trans-Valerenyl acetate | C_17_H_26_O_2_ | 0.0895 | 0.0778 | 0.0701 | 0.0742 |

Table S6 Results of HS-GC-MS for AMR (Continued)

| Serial Number | Retention Time | Compound | Molecular formula | Average Relative Content（%） | | | |
| --- | --- | --- | --- | --- | --- | --- | --- |
|  |  |  |  | ZJ | AH-HN-1 | AH-HN-2 | HB |
| 44 | 34.556 | 2-Methylheptanoic acid | C_8_H_16_O_2_ | 0.0048 | 0.0027 | 0.0007 | 0.0060 |
| 45 | 34.764 | (E)-Valerenyl isovalerate | C_20_H_32_O_2_ | 0.0080 | 0.0034 | 0.0015 | 0.0025 |
| 46 | 36.545 | Naphtho[2,3-b]furan-2(3H)-one, 4a,5,6,7,8,8a-hexahydro-3,8a-dimethyl-5-methylene- | C_15_H_18_O_2_ | 0.0157 | 0.0251 | 0.0148 | 0.0109 |
| 47 | 37.156 | Oxalic acid, hexyl 2-methylphenyl ester | C_15_H_20_O_4_ | 0.0065 | 0.0067 | 0.0000 | 0.0027 |
| 48 | 37.666 | Naphtho[2,3-b]furan-2(4H)-one, 4a,5,6,7,8,8a,9,9a-octahydro-3,8a-dimethyl-5-methylene- | C_15_H_20_O_2_ | 0.0033 | 0.0100 | 0.0054 | 0.0019 |
| 49 | 38.034 | 1,4-Methanocycloocta[d]pyridazine, 1,4,4a,5,6,9,10,10a-octahydro-11,11-dimethyl-, (1.α.,4.α.,4a.α.,10a.α.)- | C_13_H_20_N_2_ | 0.0307 | 0.0508 | 0.0386 | 0.0253 |
| 50 | 39.31 | 1-Tridecyne | C_13_H_24_ | 0.0103 | 0.0276 | 0.0167 | 0.0364 |

## Table S7

Table S7 Identification Results of Chemical Components in AMR

| Serial Number | *t_R_*/min | Molecular formula | Sum Formula | Theoretical（*m/z*） | Measured（*m/z*） | Error  （×10^-6^） | Compound | MS/MS Fragments |
| --- | --- | --- | --- | --- | --- | --- | --- | --- |
| 1 | 0.74 | C_21_H_29_O_11_ | M+H | 458.1783 | 458.1755 | -6.06 | (E)-deca-2-ene-4,-diyne-1,10-diol-1-O-β-Dapiofuranosyl-(1→6)-β-D-glu-copyranoside | 116.0936、70.0795 |
| 2 | 0.94 | C_12_H_22_O_11_ | M-H | 341.1089 | 341.1112 | 6.74 | Sucrose | 113.0245、101.0243、89.0244、85.0293、73.0295、71.0138、59.0138 |
| 3 | 1.28 | C_6_H_14_N_4_O_2_ | M+H | 174.1117 | 175.1181 | 1.33 | L-Arginine | 158.1229、130.1187、116.0946 |
| 4 | 1.49 | C_9_H_12_N_2_O_6_ | M-H | 243.0623 | 243.0615 | -3.29 | Uridine | 200.0566、152.0352、110.0246 |
| 5 | 2.04 | C_12_H_22_O_11_ | M+H | 343.1235 | 343.1227 | 2.33 | D-(+)-Maltose | 269.0537、181.2071、165.0542、145.0493、103.0391、85.0283、75.0631 |
| 6 | 2.51 | C8H16N4O3 | M+H | 217.1291 | 217.1290 | -2.46 | Acetylarginine | 217.1291，158.0809，112.0867 |
| 7 | 3.26 | C_16_H_18_O_9_ | M-H | 353.0878 | 353.0874 | -1.13 | Neochlorogenic acid | 191.0560、179.0349、135.0451 |
| 8 | 3.47 | C_22_H_38_O_12_ | M-H | 493.229 | 493.2281 | -1.82 | (1R,4S,6R)-1,3,3-trimethyl-2-oxabicyclo [2.2.2] oct-6-yl-6-O-β-D-glucopyranosyl-β-D-glucopyranoside | 447.2231、285.1703、161.0450、143.0345、119.0349、101.0242、89.0243、71.0137、59.0138 |
| 9 | 3.5 | C_11_H_12_N_2_O_2_ | M+H | 205.0972 | 205.0964 | -3.90 | DL-Tryptophan | 159.0914、142.0646、132.0805、130.0648 |

Table S7 Identification Results of Chemical Components in AMR(Continued)

| Serial Number | *t_R_*/min | Molecular formula | Sum Formula | Theoretical（*m/z*） | Measured（*m/z*） | Error  （×10^-6^） | Compound | MS/MS Fragments |
| --- | --- | --- | --- | --- | --- | --- | --- | --- |
| 10 | 3.73 | C_13_H_16_O_8_ | M-H | 299.0782 | 299.0769 | -1.02 | 4-(beta-D-Glucosyloxy)benzoate | 137.0243，93.0344，138.0276 |
| 11 | 3.95 | C_11_H_12_O_6_ | M-H | 239.0560 | 239.0561 | -0.17 | (1R_6R)-6-Hydroxy-2-succinylcyclohexa-2_4-diene-1-carboxylate | 179.0347，149.0608，239.0561 |
| 12 | 4.14 | C_18_H_32_O_16_ | M+H | 505.1763 | 505.1773 | 1.98 | Melezitose | 145.0494、127.0388、85.0280 |
| 13 | 4.36 | C_25_H_24_O_12_ | M-H | 515.1195 | 515.1194 | -0.19 | Isochlorogenic acid B | 353.0870、191.0561、173.0450 |
| 14 | 4.99 | C_23_H_28_O_11_ | M+H | 498.1969 | 498.1971 | 0.40 | Paeoniflorin | 179.0700，151.0752，180.0734 |
| 15 | 5.69 | C_16_H_18_O_9_ | M-H | 353.0878 | 353.0869 | -2.55 | Chlorogenic acid | 191.0560、179.0348、135.0452 |
| 16 | 5.79 | C_25_H_24_O_12_ | M-H | 515.1195 | 515.1189 | -1.16 | Isochlorogenic acid A | 353.0889、191.0561、179.0349、135.0450 |
| 17 | 5.97 | C_25_H_24_O_12_ | M-H | 515.1195 | 515.1187 | -1.55 | Isochlorogenic acid C | 353.0870、191.0561、179.0349、173.0450、135.0451 |
| 18 | 6.1 | C_36_H_70_O_11_ | M+H | 679.4991 | 679.5009 | 2.65 | glucopyranoside | 661.4996、452.3588、435.3322、209.1644 |
| 19 | 6.56 | C_11_H_10_O_4_ | M+H | 207.0652 | 207.0648 | -1.93 | Scoparone | 191.0339、163.1117、147.1167、133.1010、119.0854、107.0851 |
| 20 | 7.33 | C_18_H_22_O_6_ | M-H | 333.1344 | 333.1342 | -0.60 | 6-(3-hydroxy-propionyloxy) atractylenolid III | 305.1389、261.1497、244.1420 |

Table S7 Identification Results of Chemical Components in AMR(Continued)

| Serial Number | *t_R_*/min | Molecular formula | Sum Formula | Theoretical（*m/z*） | Measured（*m/z*） | Error  （×10^-6^） | Compound | MS/MS Fragments |
| --- | --- | --- | --- | --- | --- | --- | --- | --- |
| 21 | 8.03 | C_20_H_36_O_7_ | M+H | 389.2534 | 389.2535 | 0.26 | 8,9-epoxy atracolactone | 371.2438、330.2047、232.1696、145.8874、70.0652 |
| 22 | 8.73 | C_15_H_22_O_5_ | M-H | 281.1394 | 281.1396 | 0.71 | 4,6-Dihydroxy-3,3a-dihydroatractylenolide III | 263.1297、245.1200 |
| 23 | 8.85 | C_15_H_22_O_5_ | M-H | 281.1394 | 281.1392 | -0.71 | 6,9-Dihydroxy-3,3a-dihydroatractylenolide III | 263.1297、245.1200 |
| 24 | 9.14 | C_12_H_14_O_4_ | M-H | 221.0819 | 221.0817 | -0.90 | Monobutyl phthalate | 147.0085、121.0295、93.0343、75.0008 |
| 25 | 9.56 | C_16_H_20_O | M+H | 229.1587 | 229.1583 | -1.75 | furan sesquiterpene | 214.1352、173.0964、133.0646、121.0645 |
| 26 | 9.76 | C_15_H_22_O_3_ | M-H | 249.1496 | 249.1496 | 0 | 2-(3-hydroxy-4a-methyl-8-methylene-octahydronaphthalen-2-ylidene)-propionic aci | 231.1390、205.1598 |
| 27 | 10.67 | C_15_H_20_O_3_ | M+H | 249.1485 | 249.1478 | -2.81 | 6-hydroxyatractylenolide I | 231.1377、213.1268、203.1424 |
| 28 | 10.97 | C_15_H_19_NO | M+H | 230.1539 | 230.1536 | -1.30 | Atractylenolactam | 185.1333、160.1757 |
| 29 | 11.19 | C_29_H_50_O | M+H | 415.3934 | 415.3924 | -2.41 | β- Sitosterol | 119.0854 |
| 30 | 11.70 | C_18_H_39_ NO_3_ | M+H | 318.2997 | 318.2994 | -2.74 | 2-Amino-1,3,4-octadecanetriol | 60.0446，282.2785，300.2892 |
| 31 | 11.93 | C_15_H_20_O_2_ | M+H | 233.1536 | 233.1534 | -0.86 | atractylenolide I | 215.1422、187.1480、159.0800、145.1018、131.0854、105.0696、95.0852 |

Table S7 Identification Results of Chemical Components in AMR(Continued)

| Serial Number | *t_R_*/min | Molecular formula | Sum Formula | Theoretical（*m/z*） | Measured（*m/z*） | Error  （×10^-6^） | Compound | MS/MS Fragments |
| --- | --- | --- | --- | --- | --- | --- | --- | --- |
| 32 | 12.07 | C_15_H_20_O_2_ | M+H | 233.1536 | 233.1531 | -2.14 | isoatractylode A | 215.1426、187.1477、159.1166 |
| 33 | 12.21 | C_16_H_16_O_3_ | M+H | 257.1172 | 257.1168 | -1.56 | P-hydroxyphenyl ethyl phenylacetate | 178.0775、165.0700、153.0697、141.0692 |
| 34 | 12.74 | C_18_H_32_O_3_ | M-H | 295.2279 | 295.2277 | -0.68 | 9,10-epoxy-12(Z)-octadecenoic acid | 171.1025 |
| 35 | 12.97 | C_18_H_32_O_3_ | M-H | 295.2279 | 295.2277 | -0.68 | 1-(4-hydroxy-pentyl)-2,8a-dimethyl-5-methylenedecahydro-naphthalene-2,6-diol | 277.2171、171.1022 |
| 36 | 13.23 | C_15_H_24_ | M+H | 205.1951 | 205.1949 | -0.97 | β- Farnesene | 149.1324、135.1167、123.1166、95.0854 |
| 37 | 13.37 | C_31_H_45_NO_4_ | M+H | 496.3421 | 496.3401 | -4.03 | 7-[4-(11-hydroxy-undecyloxy)-phenyl]-7-pyridin-3-ylhept-6-enoic acid ethyl ester | 478.3292、184.0731、104.1068 |
| 38 | 13.63 | C_17_H_22_O_3_ | M+H | 275.1642 | 275.1637 | -1.82 | 3β-Acetoxyatractylone | 215.1428、145.1010、119.0854、105.0698、91.0542 |
| 39 | 13.97 | C_15_H_18_O_2_ | M+H | 231.138 | 231.1374 | -2.60 | atractylenolide II | 213.1271、185.1321、157.1008、91.0538 |
| 40 | 14.35 | C_15_H_22_O | M+H | 219.1743 | 219.1737 | -2.74 | Atractylmacrol D | 201.1632、173.1319、107.0853 |
| 41 | 14.78 | C_16_H_22_O_3_ | M+H | 263.1642 | 263.1634 | -3.04 | 8β-Methoxyatractylenolide I | 163.1478、105.0695 |
| 42 | 15.18 | C_34_H_68_O_19_ | M+H | 781.4428 | 781.4363 | -8.32 | rotanane heptaecanate | 499.2880、295.1535、211.0960、85.0646 |

Table S7 Identification Results of Chemical Components in AMR(Continued)

| Serial Number | *t_R_*/min | Molecular formula | Sum Formula | Theoretical（*m/z*） | Measured（*m/z*） | Error  （×10^-6^） | Compound | MS/MS Fragments |
| --- | --- | --- | --- | --- | --- | --- | --- | --- |
| 43 | 15.37 | C_15_H_22_ | M+H | 203.1794 | 203.179 | -1.97 | Curcumene | 159.1169、147.1165、133.1009、119.0853 |
| 44 | 15.43 | C_15_H_22_ | M+H | 203.1794 | 203.1791 | -1.48 | atractylenolideⅥ | 161.1322、133.1010、105.0698 |
| 45 | 15.50 | C_15_H_20_O | M+H | 217.1587 | 217.1584 | -1.38 | atractylone | 199.1479、159.1167、133.1010 |
| 46 | 15.60 | C_15_H_22_ | M+H | 203.1794 | 203.1792 | -0.98 | β-vatirenene | 161.1324、147.1166、133.1010、121.1010 |
| 47 | 15.66 | C_15_H_22_ | M+H | 203.1794 | 203.1792 | -0.98 | Deoxyaromadendrene | 161.1324、147.1166、133.1010、121.1010 |
| 48 | 15.80 | C_18_H_35_NO | M+H | 282.2791 | 282.2785 | -2.13 | Oleamide | 123.1165、109.1009、97.1010、83.0853、79.0541、69.0698 |
| 49 | 16.09 | C_30_H_38_O_4_ | M+H | 463.2843 | 463.2846 | 0.65 | Biatractylolide | 231.1378、163.1115 |
| 50 | 16.81 | C_15_H_22_O | M+H | 219.1743 | 219.174 | -1.37 | Selinadienone | 201.1635、159.1166、145.1011、131.0855、123.0805、105.0697、95.0854 |
| 51 | 17.74 | C_22_H_43_NO | M+H | 338.3417 | 338.3408 | -2.66 | Erucamide | 212.2008、184.1696、153.1269、123.1166、109.1009、97.1010、 |

## Table S8

Table S8 Spearman correlation analysis between sensory evaluation scores and differential components

|  | caryophyllene | γ-elemene | aromadendrene | atractylone | isoatractylode A | β-sitosterol | atractylenolide Ⅰ | maltose | sucrose | 6,9-dihydroxy-3,3a-dihydro atractylenolide III |
| --- | --- | --- | --- | --- | --- | --- | --- | --- | --- | --- |
| sensory evaluation scores | 0.342* | 0.332* | 0.318* | 0.191 | 0.296 | -0.030 | 0.604** | -0.034 | -0.408** | 0.139 |

^*^*P* < 0.05, ^**^*P* < 0.01.

## Table S9

Table S9 Spearman correlation analysis of E-nose and HS-GC-MS compounds

| Compounds | S1 | S5 | S7 | S11 | S12 | S13 | S14 | S17 | S18 |
| --- | --- | --- | --- | --- | --- | --- | --- | --- | --- |
| cpd7 | -0.016 | 0.270 | -0.390* | -0.362* | -0.376* | -0.410** | -0.372* | -0.162 | -0.222 |
| cpd8 | -0.006 | -0.283 | 0.196 | 0.241 | 0.241 | 0.161 | 0.215 | 0.347* | 0.352* |
| cpd9 | 0.221 | -0.383* | 0.199 | 0.200 | 0.224 | 0.178 | 0.202 | 0.128 | 0.158 |
| cpd10 | 0.072 | -0.317* | 0.206 | 0.238 | 0.247 | 0.184 | 0.222 | 0.274 | 0.283 |
| cpd11 | 0.144 | -0.352* | 0.229 | 0.257 | 0.266 | 0.215 | 0.247 | 0.232 | 0.247 |
| cpd13 | 0.502** | -0.004 | -0.064 | -0.108 | -0.102 | -0.122 | -0.089 | -0.118 | -0.116 |
| cpd14 | 0.000 | -0.316* | 0.235 | 0.293 | 0.286 | 0.200 | 0.254 | 0.414** | 0.403* |
| cpd15 | 0.468** | 0.002 | 0.003 | -0.051 | -0.041 | -0.048 | -0.029 | -0.101 | -0.109 |
| cpd20 | 0.553** | -0.223 | 0.164 | 0.145 | 0.138 | 0.137 | 0.145 | -0.019 | 0.011 |
| cpd29 | -0.132 | 0.107 | 0.045 | 0.046 | 0.049 | -0.021 | 0.031 | 0.331* | 0.280 |
| cpd35 | -0.155 | -0.018 | 0.077 | 0.134 | 0.104 | 0.059 | 0.080 | 0.356* | 0.336* |
| cpd38 | 0.329* | 0.007 | -0.070 | -0.097 | -0.086 | -0.110 | -0.081 | -0.085 | -0.073 |
| cpd42 | -0.020 | -0.054 | 0.096 | 0.131 | 0.124 | 0.068 | 0.091 | 0.369* | 0.342* |
| cpd44 | 0.099 | 0.094 | -0.172 | -0.268 | -0.245 | -0.163 | -0.233 | -0.524** | -0.479** |
| cpd45 | 0.078 | 0.240 | -0.258 | -0.334* | -0.325* | -0.242 | -0.322* | -0.288 | -0.275 |
| cpd48 | -0.197 | 0.104 | -0.056 | -0.018 | -0.015 | -0.088 | -0.062 | 0.320* | 0.252 |
| cpd50 | -0.361* | 0.166 | -0.189 | -0.175 | -0.145 | -0.157 | -0.210 | -0.040 | -0.091 |

^*^*P* < 0.05, ^**^*P* < 0.01.

## Table S10

Table S10 Spearman correlation analysis of E-tongue and LC-MS compounds

| Compounds | ANS | PKS | Compounds | ANS | PKS | Compounds | ANS | PKS |
| --- | --- | --- | --- | --- | --- | --- | --- | --- |
| Peak1 | -0.324* | -0.213 | Peak18 | 0.124 | -0.091 | Peak35 | -0.324* | -0.163 |
| Peak2 | 0.635** | 0.603** | Peak19 | -0.005 | 0.132 | Peak36 | 0.140 | 0.179 |
| Peak3 | 0.347* | 0.447** | Peak20 | 0.419** | 0.109 | Peak37 | -0.465** | -0.299 |
| Peak4 | 0.332* | 0.364* | Peak21 | -0.374* | -0.351* | Peak38 | 0.221 | 0.329* |
| Peak5 | -0.206 | -0.052 | Peak22 | -0.034 | -0.048 | Peak39 | -0.153 | -0.276 |
| Peak6 | -0.111 | -0.019 | Peak23 | -0.170 | -0.129 | Peak40 | -0.036 | -0.197 |
| Peak7 | 0.393* | 0.117 | Peak24 | 0.117 | -0.003 | Peak41 | 0.094 | -0.047 |
| Peak8 | -0.276 | -0.214 | Peak25 | 0.185 | -0.169 | Peak42 | 0.439** | 0.052 |
| Peak9 | -0.384* | -.450** | Peak26 | 0.128 | 0.069 | Peak43 | -0.067 | -0.021 |
| Peak10 | 0.425** | 0.199 | Peak27 | -0.370* | -0.255 | Peak44 | -0.002 | -0.105 |
| Peak11 | 0.460** | 0.037 | Peak28 | 0.139 | -0.187 | Peak45 | -0.254 | -0.314 |
| Peak12 | -0.010 | -0.246 | Peak29 | -0.155 | 0.086 | Peak46 | 0.174 | 0.178 |
| Peak13 | 0.467** | 0.251 | Peak30 | -0.343* | -0.021 | Peak47 | 0.348* | 0.158 |
| Peak14 | 0.019 | 0.000 | Peak31 | -0.248 | -0.483** | Peak48 | 0.089 | -0.023 |
| Peak15 | 0.416** | 0.272 | Peak32 | -0.266 | -0.308 | Peak49 | -0.183 | 0.106 |
| Peak16 | -0.025 | -0.293 | Peak33 | -0.009 | 0.002 | Peak50 | 0.304 | -0.002 |
| Peak17 | 0.206 | 0.180 | Peak34 | -0.417** | -0.201 | Peak51 | 0.327* | -0.276 |

^*^*P* < 0.05, ^**^*P* < 0.01.

## Figure S1


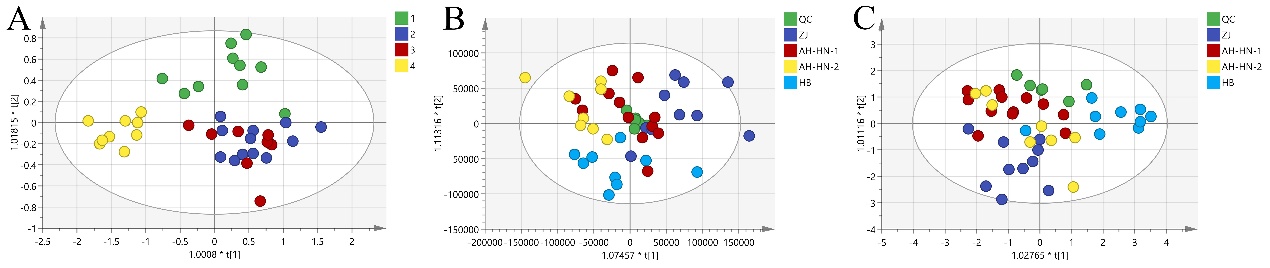


Figure S1 OPLS-DA score plot (A: HS-GC-MS, 1: ZJ; 2: AH-HN-A; 3: AH-HN-2; 4: HB. B: LC-MS analysis of positive ion mode; C: LC-MS analysis of negative ion mode)
